# Supplementary material for: Affinity-optimizing enhancer variants disrupt development
Source: Nature. 2024 Jan 17;626(7997):151–9. doi: 10.1038/s41586-023-06922-8 (PMC10830414; doi:10.1038/s41586-023-06922-8)

nature portfolio
https://doi.org/10.1038/s41586-023-06922-8

Supplementary information

Affinity-optimizing enhancer variants disrupt development

In the format provided by the authors and unedited

Supplementary Information Figure 1

A ETS-1 binding to ETS-A site in ZRS
(Extended Data Figure 4A)

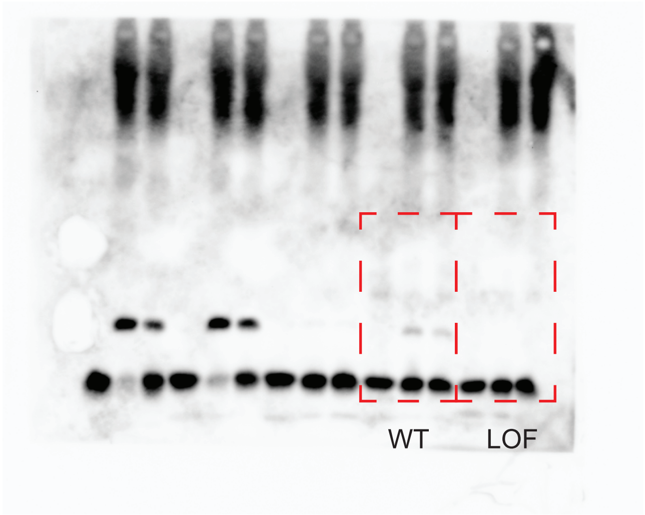

B ETS-1 binding to ETS-A site in ZRS
(Extended Data Figure 4B)

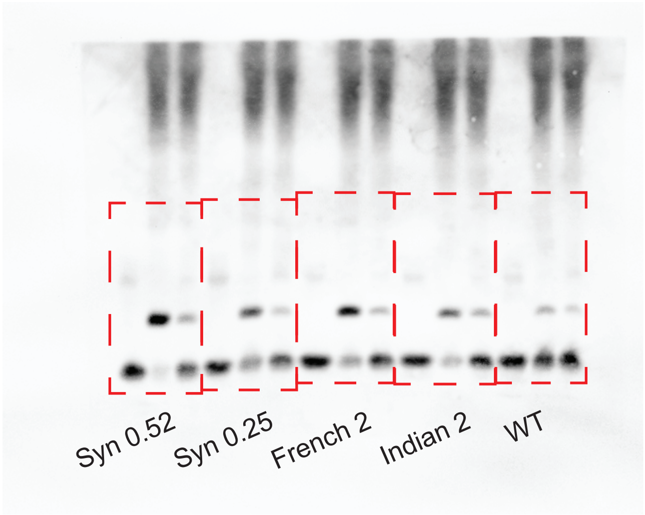

C HOXA13 binding to Dutch 2 variant in ZRS
(Extended Data Figure 8A)

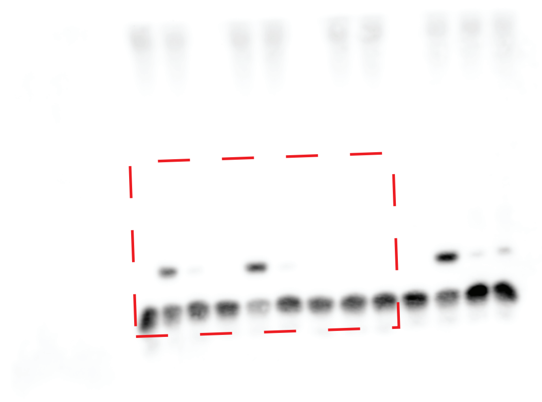

D HOXD13 binding to Dutch 2 variant in ZRS
(Extended Data Figure 8B)

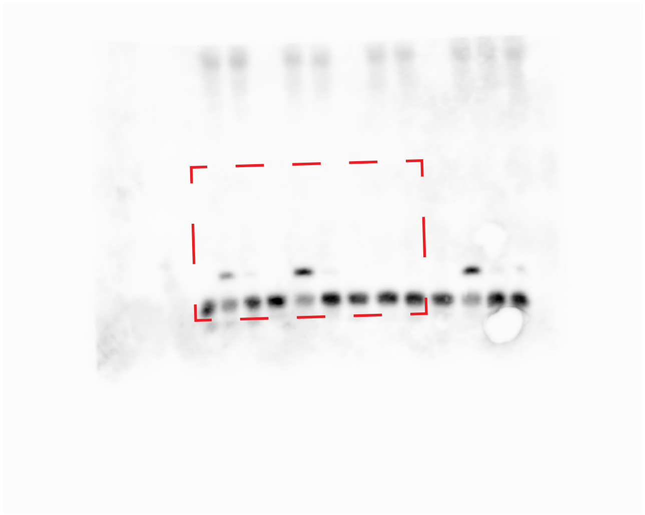

Supplement: Supplementary file 1 — Raw EMSA images for Extended Data Figures 4 and 8. [file 41586_2023_6922_MOESM1_ESM.pdf]
