# Supplementary material for: Affinity-optimizing enhancer variants disrupt development
Source: Nature. 2024 Jan 17;626(7997):151–9. doi: 10.1038/s41586-023-06922-8 (PMC10830414; doi:10.1038/s41586-023-06922-8)
Supplement: Supplementary file 2 — Reporting Summary [file 41586_2023_6922_MOESM2_ESM.pdf]

nature portfolio

Corresponding author(s): Emma Farley

Last updated by author(s): Sep 26, 2023

Reporting Summary

Nature Portfolio wishes to improve the reproducibility of the work that we publish. This form provides structure for consistency and transparency in reporting. For further information on Nature Portfolio policies, see our Editorial Policies and the Editorial Policy Checklist.

Statistics

For all statistical analyses, confirm that the following items are present in the figure legend, table legend, main text, or Methods section.

n/a	Confirmed	
☐	☒	The exact sample size (*n*) for each experimental group/condition, given as a discrete number and unit of measurement
☐	☒	A statement on whether measurements were taken from distinct samples or whether the same sample was measured repeatedly
☐	☒	The statistical test(s) used AND whether they are one- or two-sided<br>*Only common tests should be described solely by name; describe more complex techniques in the Methods section.*
☒	☐	A description of all covariates tested
☐	☒	A description of any assumptions or corrections, such as tests of normality and adjustment for multiple comparisons
☐	☒	A full description of the statistical parameters including central tendency (e.g. means) or other basic estimates (e.g. regression coefficient) AND variation (e.g. standard deviation) or associated estimates of uncertainty (e.g. confidence intervals)
☐	☒	For null hypothesis testing, the test statistic (e.g. *F*, *t*, *r*) with confidence intervals, effect sizes, degrees of freedom and *P* value noted<br>*Give P values as exact values whenever suitable.*
☒	☐	For Bayesian analysis, information on the choice of priors and Markov chain Monte Carlo settings
☒	☐	For hierarchical and complex designs, identification of the appropriate level for tests and full reporting of outcomes
☒	☐	Estimates of effect sizes (e.g. Cohen's *d*, Pearson's *r*), indicating how they were calculated

Our web collection on statistics for biologists contains articles on many of the points above.

Software and code

Policy information about availability of computer code

Data collection	No custom code was used in data collection.
Data analysis	Standard python libraries were used to analyze MPRA, ChIP and eQTL data (pandas 1.4.2 , numpy 1.22.3, statsmodels 0.13.2, seaborn 0.11.2 , bx 0.9.0, pyliftover 0.4, Bio 1.79, scipy, matplotlib). Custom python code utilizing these libraries were used to process, visualize and do statistics. Python version 3.9.7 was used. ImageLab Software Version 6.1 was used for EMSA analysis.

For manuscripts utilizing custom algorithms or software that are central to the research but not yet described in published literature, software must be made available to editors and reviewers. We strongly encourage code deposition in a community repository (e.g. GitHub). See the Nature Portfolio guidelines for submitting code & software for further information.

Data

Policy information about availability of data

All manuscripts must include a data availability statement. This statement should provide the following information, where applicable:

- Accession codes, unique identifiers, or web links for publicly available datasets
- A description of any restrictions on data availability
- For clinical datasets or third party data, please ensure that the statement adheres to our policy

All data supporting the findings of this study are available within the paper and its Supplementary Information. ChIP-Seq data analyzed in this paper were downloaded using accession codes GSM2218592, GSM3520734, GSM4110116. eQTL data analyzed in this paper was downloaded from the EBI eQTL catalogue

Github page (https://github.com/eQTL-Catalogue/eQTL-Catalogue-resources/blob/master/tabix/) for lymphoblastoid cell line generated by Geuvadis consortium. UniProbe database (http://thebrain.bwh.harvard.edu/pbms/UniPROBE_staging/browse.php) was used to access PBM data.

Research involving human participants, their data, or biological material

Policy information about studies with human participants or human data. See also policy information about sex, gender (identity/presentation), and sexual orientation and race, ethnicity and racism.

Reporting on sex and gender	Not applicable
Reporting on race, ethnicity, or other socially relevant groupings	Not applicable
Population characteristics	Not applicable
Recruitment	Not applicable
Ethics oversight	Not applicable

Note that full information on the approval of the study protocol must also be provided in the manuscript.

Field-specific reporting

Please select the one below that is the best fit for your research. If you are not sure, read the appropriate sections before making your selection.

☒ Life sciences ☐ Behavioural & social sciences ☐ Ecological, evolutionary & environmental sciences

For a reference copy of the document with all sections, see nature.com/documents/nr-reporting-summary-flat.pdf

Life sciences study design

All studies must disclose on these points even when the disclosure is negative.

Sample size	We sought to study as many mice as possible, we stopped when we had around 80 mice for each genotype for heterozygotes and close to 40 for homozygotes.
Data exclusions	We have not excluded any data.
Replication	We analyzed mice from multiple litters and repeated all experiments at least twice with similar results.
Randomization	We did not need to do this for our experimental design, since there are no applicable covariates to randomize for in this study.
Blinding	Each mouse born into our colony has all 4 limbs inspected by an investigator blind to genotype at postnatal day 10-18 during routine ear clipping (for identification) and tail biopsy collection (for genotyping).

Reporting for specific materials, systems and methods

We require information from authors about some types of materials, experimental systems and methods used in many studies. Here, indicate whether each material, system or method listed is relevant to your study. If you are not sure if a list item applies to your research, read the appropriate section before selecting a response.

Materials & experimental systems

n/a	Involved in the study	
☐	☒	Antibodies
☒	☐	Eukaryotic cell lines
☒	☐	Palaeontology and archaeology
☐	☒	Animals and other organisms
☒	☐	Clinical data
☒	☐	Dual use research of concern
☒	☐	Plants

Methods

n/a	Involved in the study	
☒	☐	ChIP-seq
☒	☐	Flow cytometry
☒	☐	MRI-based neuroimaging

Antibodies

Antibodies used	1:2500 anti-digoxigenin-AP antibody (Roche, 11093274910)
Validation	Other papers that have also used this antibody and found it effective: PMID33412105, PMID36184733, PMID30945286

Animals and other research organisms

Policy information about studies involving animals; ARRIVE guidelines recommended for reporting animal research, and Sex and Gender in Research

Laboratory animals	Mice C57Bl/6NHsd, mice were phenotyped at 10-18 days old. Mice are housed at room temperature (between 68-79°F) and regular humidity. Since housing temperature and humidity are ambient and not relevant to the study, we did not include this information in the manuscript. All other relevant housing conditions for mice is included in the methods section.
Wild animals	The study did not involve wild animals.
Reporting on sex	Mice of both sexes were involved in this study. Sex is determined during 10-21 days old by comparison of urogenital-anal distance and presence of female nipples or male genitalia. Phenotypes of mice based on sex is reported in Supplementary Materials.
Field-collected samples	This study did not involve samples collected from the field.
Ethics oversight	All animal procedures and studies were approved by the University of California, San Diego Institutional Animal Care and Use Committee according to the Association for Assessment and Accreditation of Laboratory Animal Care guidelines.

Note that full information on the approval of the study protocol must also be provided in the manuscript.
