# Supplementary material for: Affinity-optimizing enhancer variants disrupt development
Source: Nature. 2024 Jan 17;626(7997):151–9. doi: 10.1038/s41586-023-06922-8 (PMC10830414; doi:10.1038/s41586-023-06922-8)
Supplement: Supplementary file 8 — Supplementary References [file 41586_2023_6922_MOESM8_ESM.docx]

**Additional References**

52. Cooper, K. L. *et al.* Patterning and post-patterning modes of evolutionary digit loss in mammals. *Nature* **511**, 41–45 (2014).

53. Bulyk, M. L. Protein Binding Microarrays for the Characterization of DNA–Protein Interactions. in *Analytics of Protein–DNA Interactions* (ed. Seitz, H.) 65–85 (Springer, 2007). doi:10.1007/10_025.

54. Wasylyk, C., Gutman, A., Nicholson, R. & Wasylyk, B. The c-Ets oncoprotein activates the stromelysin promoter through the same elements as several non-nuclear oncoproteins. *EMBO J* **10**, 1127–1134 (1991).

55. Lamber, E. P. *et al.* Regulation of the transcription factor Ets-1 by DNA-mediated homo-dimerization. *EMBO J* **27**, 2006–2017 (2008).

56. Fonseca, G. J. *et al.* Diverse motif ensembles specify non-redundant DNA binding activities of AP-1 family members in macrophages. *Nat Commun* **10**, 414 (2019).

57. Panne, D., Maniatis, T. & Harrison, S. C. An atomic model of the interferon-beta enhanceosome. *Cell* **129**, 1111–1123 (2007).

58. Saelee, P., Kearly, A., Nutt, S. L. & Garrett-Sinha, L. A. Genome-Wide Identification of Target Genes for the Key B Cell Transcription Factor Ets1. *Frontiers in Immunology* **8**, (2017).

59. Taveirne, S. *et al.* The transcription factor ETS1 is an important regulator of human NK cell development and terminal differentiation. *Blood* **136**, 288–298 (2020).

60. McCarter, A. C. *et al.* Combinatorial ETS1-Dependent Control of Oncogenic NOTCH1 Enhancers in T-cell Leukemia. *Blood Cancer Discovery* **1**, 178–197 (2020).

61. Pufall, M. A. *et al.* Variable Control of Ets-1 DNA Binding by Multiple Phosphates in an Unstructured Region. *Science* **309**, 142–145 (2005).

62. Wang, Y. *et al.* Analysis of the 2.0 Å Crystal Structure of the Protein−DNA Complex of the Human PDEF Ets Domain Bound to the Prostate Specific Antigen Regulatory Site,. *Biochemistry* **44**, 7095–7106 (2005).

63. Nitta, K. R. *et al.* Conservation of transcription factor binding specificities across 600 million years of bilateria evolution. *eLife* **4**, e04837 (2015).

64. Badis, G. *et al.* Diversity and Complexity in DNA Recognition by Transcription Factors. *Science* **324**, 1720–1723 (2009).

65. Heutink, P. *et al.* The gene for triphalangeal thumb maps to the subtelomeric region of chromosome 7q. *Nat Genet* **6**, 287–292 (1994).

66. Zhao, X., Yang, W., Sun, M. & Zhang, X. [ZRS mutations in two Chinese Han families featuring triphalangeal thumbs and preaxial polydactyly]. *Zhonghua Yi Xue Yi Chuan Xue Za Zhi* **33**, 281–285 (2016).

67. Baas, M. *et al.* Intrafamilial variability of the triphalangeal thumb phenotype in a Dutch population: Evidence for phenotypic progression over generations? *American Journal of Medical Genetics Part A* **173**, 2898–2905 (2017).

68. Zhang, Z., Lyu, Y., Li-Ling, J. & Liu, C. [Mutation analysis in a large Chinese pedigree affected with preaxial polydactyly II]. *Zhonghua Yi Xue Yi Chuan Xue Za Zhi* **36**, 610–612 (2019).

69. Cai, F. *et al.* [Genetic analysis of one family with congenital limb malformations]. *Zhonghua Yi Xue Yi Chuan Xue Za Zhi* **36**, 890–892 (2019).

70. Zeng, L. *et al.* ZPA Regulatory Sequence Variants in Chinese Patients With Preaxial Polydactyly: Genetic and Clinical Characteristics. *Frontiers in Pediatrics* **10**, (2022).

71. Dorshorst, B., Okimoto, R. & Ashwell, C. Genomic regions associated with dermal hyperpigmentation, polydactyly and other morphological traits in the Silkie chicken. *J Hered* **101**, 339–350 (2010).

72. VanderMeer, J. E. *et al.* A novel ZRS mutation in a Balochi tribal family with triphalangeal thumb, pre-axial polydactyly, post-axial polydactyly, and syndactyly. *Am J Med Genet A* **158A**, 2031–2035 (2012).

73. Furniss, D. *et al.* A variant in the sonic hedgehog regulatory sequence (ZRS) is associated with triphalangeal thumb and deregulates expression in the developing limb. *Human Molecular Genetics* **17**, 2417–2423 (2008).

74. Semerci, C. N. *et al.* Homozygous feature of isolated triphalangeal thumb-preaxial polydactyly linked to 7q36: no phenotypic difference between homozygotes and heterozygotes. *Clin Genet* **76**, 85–90 (2009).

75. Álvarez, L. F. G. *et al.* A large, ten-generation family with autosomal dominant preaxial polydactyly/triphalangeal thumb: Historical, clinical, genealogical, and molecular studies. *American Journal of Medical Genetics Part A* **191**, 100–107 (2023).

76. VanderMeer, J. E. *et al.* A novel ZRS mutation leads to preaxial polydactyly type 2 in a heterozygous form and Werner mesomelic syndrome in a homozygous form. *Hum Mutat* **35**, 945–948 (2014).

77. Girisha, K. M. *et al.* A novel mutation (g.106737G>T) in zone of polarizing activity regulatory sequence (ZRS) causes variable limb phenotypes in Werner mesomelia. *Am J Med Genet A* **164A**, 898–906 (2014).

78. Zguricas, J. *et al.* Clinical and genetic studies on 12 preaxial polydactyly families and refinement of the localisation of the gene responsible to a 1.9 cM region on chromosome 7q36. *J Med Genet* **36**, 32–40 (1999).

79. Norbnop, P., Srichomthong, C., Suphapeetiporn, K. & Shotelersuk, V. ZRS 406A>G mutation in patients with tibial hypoplasia, polydactyly and triphalangeal first fingers. *J Hum Genet* **59**, 467–470 (2014).

80. Wieczorek, D. *et al.* A specific mutation in the distant sonic hedgehog (SHH) cis-regulator (ZRS) causes Werner mesomelic syndrome (WMS) while complete ZRS duplications underlie Haas type polysyndactyly and preaxial polydactyly (PPD) with or without triphalangeal thumb. *Hum Mutat* **31**, 81–89 (2010).

81. Cho, T.-J. *et al.* Tibial hemimelia-polydactyly-five-fingered hand syndrome associated with a 404 G>A mutation in a distant sonic hedgehog cis-regulator (ZRS): a case report. *J Pediatr Orthop B* **22**, 219–221 (2013).

82. Masuya, H. *et al.* A series of ENU-induced single-base substitutions in a long-range cis-element altering Sonic hedgehog expression in the developing mouse limb bud. *Genomics* **89**, 207–214 (2007).

83. Zhao, J. *et al.* HnRNP U mediates the long-range regulation of Shh expression during limb development. *Human Molecular Genetics* **18**, 3090–3097 (2009).

84. Wu, P.-F. *et al.* A Novel ZRS Mutation in a Chinese Patient with Preaxial Polydactyly and Triphalangeal Thumb. *Cytogenet Genome Res* **149**, 171–175 (2016).

85. Vanlerberghe, C. *et al.* Intrafamilial variability of ZRS-associated syndrome: characterization of a mosaic ZRS mutation by pyrosequencing. *Clin Genet* **88**, 479–483 (2015).

86. Xu, C. *et al.* A novel ZRS variant causes preaxial polydactyly type I by increased sonic hedgehog expression in the developing limb bud. *Genetics in Medicine* **22**, 189–198 (2020).

87. Farooq, M. *et al.* Preaxial polydactyly/triphalangeal thumb is associated with changed transcription factor-binding affinity in a family with a novel point mutation in the long-range cis-regulatory element ZRS. *Eur J Hum Genet* **18**, 733–736 (2010).

88. Maas, S. A., Suzuki, T. & Fallon, J. F. Identification of spontaneous mutations within the long-range limb-specific Sonic hedgehog enhancer (ZRS) that alter Sonic hedgehog expression in the chicken limb mutants oligozeugodactyly and silkie breed. *Dev Dyn* **240**, 1212–1222 (2011).

89. Dunn, I. C. *et al.* The chicken polydactyly (Po) locus causes allelic imbalance and ectopic expression of Shh during limb development. *Developmental Dynamics* **240**, 1163–1172 (2011).

90. Knudsen, T. B. & Kochhar, D. M. Limb development in mouse embryos. III. Cellular events underlying the determination of altered skeletal patterns following treatment with 5’fluoro-2-deoxyuridine. *Teratology* **23**, 241–251 (1981).

91. Gurnett, C. A. *et al.* Two novel point mutations in the long-range SHH enhancer in three families with triphalangeal thumb and preaxial polydactyly. *Am J Med Genet A* **143A**, 27–32 (2007).

92. Al-Qattan, M. M., Al Abdulkareem, I., Al Haidan, Y. & Al Balwi, M. A novel mutation in the SHH long-range regulator (ZRS) is associated with preaxial polydactyly, triphalangeal thumb, and severe radial ray deficiency. *Am J Med Genet A* **158A**, 2610–2615 (2012).

93. Koyano-Nakagawa, N. *et al.* Etv2 regulates enhancer chromatin status to initiate Shh expression in the limb bud. *Nat Commun* **13**, 4221 (2022).
